# Rewriting the genome: harnessing R2 retrotransposons for precise DNA insertion

**DOI:** 10.3389/fgeed.2026.1789016

**Published:** 2026-09-04

**Authors:** Liangzheng Fu, Yachao Wu, Xiaohua Jin, Xu Ma

**Affiliations:** 1 National Human Genetic Resources Center, Beijing, China; 2 National Research Institute for Family Planning, Beijing, China; 3 State Key Laboratory of Organ Regeneration and Reconstruction, Institute of Zoology, Chinese Academy of Sciences, Beijing, China; 4 University of Chinese Academy of Sciences, Beijing, China

**Keywords:** engineering, gene editing, non-LTR retrotransposons, R2 retrotransposon, TPRT mechanism

## Abstract

CRISPR-based genome editors are fundamentally limited by their requirement for double-strand DNA breaks (DSBs), restricted transgene cargo capacity, and reliance on error-prone endogenous DNA repair mechanisms. Non–long terminal repeat (non-LTR) retrotransposons—especially the site-specific R2 element—offer a mechanistically distinct and potentially safer choice for programmable genomic integration. These elements employ target-primed reverse transcription (TPRT)—an RNA-templated integration mechanism that circumvents DSB formation and supports amplification of self-copy. This review delineates the molecular mechanism of R2 retrotransposons, emphasizing their highly specific integration into the 28 S ribosomal DNA locus—a recognized genomic safe harbor. We describe the functional domains of the R2 protein, including the reverse transcriptase, restriction-like endonuclease, and nucleic acid-binding motifs, and explain how they coordinate to achieve precise DNA cleavage and cDNA synthesis. Recent cryo–electron microscopy (cryo-EM) structures have revealed discrete RNA-protein complex that orchestrate the stepwise progression of TPRT. Informed by these mechanistic insights, researchers have engineered programmable platforms—including PRINT and STITCHR—that enable RNA-directed transgene integration in mammalian systems. These platforms establish R2 as a viable all-RNA programmable system for targeted genomic integration. Future directions include reprogramming the DNA-binding specificity of R2 through protein engineering to target loci, optimizing integration fidelity and efficiency, and mining diverse R2-like elements from metagenomic data. With continued optimization and rigorous safety validation, R2-derived platforms could supplant current nuclease-dependent editors in applications requiring high-fidelity, large-cargo integration.

## Introduction

Rapid advancements in gene therapy have accelerated the development of precise gene-editing methodologies. Gene knock-in remains a pivotal therapeutic strategy for treating various genetic disorders and modulating physiological processes. This approach typically relies on gene integration strategies that use HDR for precise editing, and DNA templates are then used to facilitate the integration of exogenous genes (Shalem et al., 2015; Wang and Doudna, 2023; Pacesa et al., 2024). However, these methods necessitate double-strand breaks (DSBs) at the target genomic sites, which can elicit heightened immunogenicity *in vivo*, lead to genomic instability, and disrupt normal cellular functions (Andrew et al., 2018; Haitao et al., 2018). The advent of CRISPR-Cas9 revolutionized genome editing by enabling programmable DNA cleavage (Jinek et al., 2012; Cong et al., 2013; Mali et al., 2013). Subsequent innovations, such as base editing (Komor et al., 2016; Gaudelli et al., 2017) and prime editing (Anzalone et al., 2019), mitigated the reliance on double-strand breaks (DSBs). Nevertheless, these approaches remain limited by modest payload capacities—less than 100 bp for base editors and roughly 100–1,000 bp for conventional prime editors—as well as inconsistent performance in primary cells (Levesque et al., 2025; Anzalone et al., 2019). In contrast, non-LTR retrotransposons integrate kilobase-scale cargoes without generating DSBs through a copy-and-paste mechanism, offering a promising route for large-gene therapy.

Transposable elements pervade eukaryotic genomes, accounting for nearly half of the human genomic landscape (Feschotte and Pritham, 2007; Wessler, 2006). Among these, non-LTR retrotransposons constitute the most abundant class and propagate through a “copy-paste” mechanism (Malik et al., 1999; Wessler, 2006; Han, 2010; Fujiwara, 2015). This process enables amplification without inducing double-strand breaks (DSBs) by relying on TPRT, which converts their RNA transcripts into genomic DNA (Han, 2010; Grandi and An, 2013; O’Donnell and Burns, 2010). As a result, retrotransposons have emerged as promising tools for efficient gene delivery, capable of integrating multiple copies of a target gene into the host genome. Based on their endonuclease domains, non-LTR retrotransposons are classified into two major types: those containing apyrimidinic/apurinic endonuclease (APE-type) domains and those with restriction-like endonuclease (RLE-type) domains (Malik et al., 1999; Wilkinson et al., 2023). A well-characterized APE-type example is the LINEs (Long Interspersed Nuclear Elements) family (Feng et al., 1996; Hohjoh and Singer, 2014; Geis and Goff, 2020). Recent studies have demonstrated stable multicopy integration of target genes using LINE-1 retrotransposons, underscoring their potential for gene integration applications (Zheng et al., 2021). However, LINEs exhibit relatively broad targeting specificity with a strong preference for AT-rich genomic regions (Thawani et al., 2023; Steranka et al., 2019), leading to substantial off-target risks that complicate engineering approaches. In contrast, beyond the well-studied LINEs, RLE-type retrotransposons include members with stringent insertion specificity. These elements encode endonucleases that recognize conserved genomic sequences and promote site-specific integration via the TPRT mechanism (Eickbush and Eickbush, 2015).

Among non-LTR retrotransposons encoding restriction-like endonuclease (RLE) domains, many are located within ribosomal RNA (rRNA) gene clusters of host genomes and are broadly categorized as R elements (Eickbush, 2007). A well-studied representative, the R2 retrotransposon, exhibits stringent integration specificity for the 28 S rDNA locus. First identified in *Drosophila melanogaster* (Dawid and Rebbert, 1981; Roiha et al., 1981), R2 has since been detected across at least six distinct animal phyla (Kenji and Kojima, 2004; Jakubczak and Eickbush, 1991; Kojima and Haruhiko, 2005; Kojima et al., 2006; Besansky et al., 1992). Distinguishing it from LINEs, R2 comprises a single open reading frame (ORF) encoding all domains required for TPRT (Eickbush, 2007). The system’s capability to recognize specific genomic sequences and achieve precise integration underscores its potential as a promising chassis for novel gene editing tools, with improved safety over existing platforms.

The distinctive properties of R2—including its site specificity, TPRT mechanism, and scalable cargo capacity—render it a compelling scaffold for next-generation genome engineering tools. Several recent studies have elucidated the molecular mechanisms underlying R2 retrotransposition and explored its applicability for gene integration in mammalian cells (Wilkinson et al., 2023; McIntyre et al., 2025; Palm et al., 2024; Deng et al., 2023; Kuroki-Kami et al., 2019; Fell et al., 2025; Zhang et al., 2024; Chen et al., 2024). This review highlights key advances in understanding R2 biology and its biotechnological potential, with the aim of guiding future efforts to exploit R2 retrotransposons for genomic evolution studies and gene therapy applications. This review will detail the molecular mechanism of R2, andsummarize the latest derived tools, and discuss future directions.

## Subsections

R2 retrotransposons have been reported in a broad range of organisms, and studies of their domain organization and parasitic strategies continue to grow (Pacesa et al., 2024). They are divided into four clades based mainly on differences in the N-terminal zinc-finger region (Luchetti and Mantovani, 2013). The N-terminal part contains a Myb domain preceded by one to three zinc fingers (ZnFs). The ZnF directly adjacent to the Myb domain is conserved and designated ZnF1; additional fingers are numbered ZnF2 and ZnF3 moving toward the N-terminus. This variability in ZnF architecture underpins a classification into four clades (further split into 11 subclades): clade A (ZnF3+ZnF2+ZnF1), clade B (ZnF2+ZnF1), clade C (ZnF3+ZnF1), and clade D (ZnF1 only) (Kojima and Haruhiko, 2005; Kojima et al., 2016). Among all characterized elements, the D-clade member R2BM from the silkworm *Bombyx mori* has been the most thoroughly investigated at the biochemical and structural levels (Anthony and Kathleen, 2024; Kova et al., 2023; Royengel, 2012). We therefore use the structure and retrotransposition pathway of the R2BM protein as a model to systematically describe the working mechanism of R2 retrotransposons.

## The structural composition of R2 retrotransposons

**FIGURE 1:**
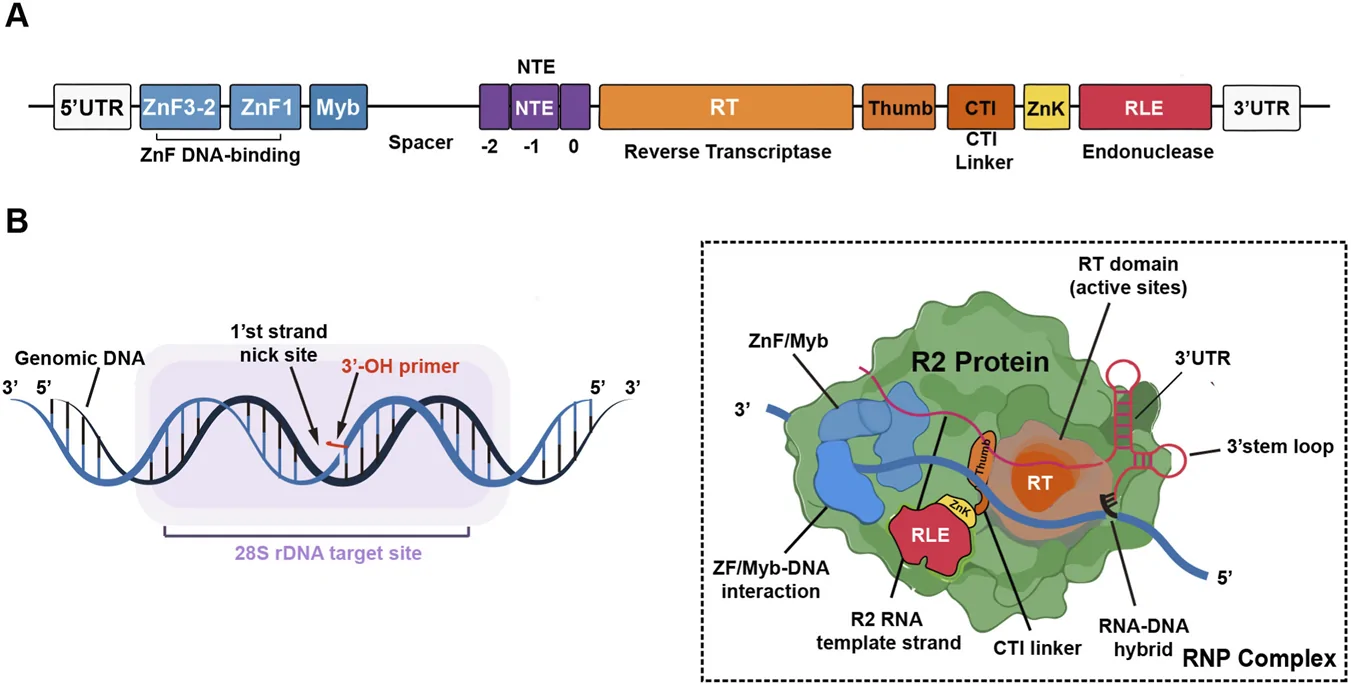
Domain architecture of the R2 retrotransposon protein and target site recognition complex. **(A)** Domain architecture of the R2 protein. The R2 protein consists of an N-terminal DNA-binding domain (comprising ZnF3-2, ZnF1, and a Myb domain), a reverse transcriptase (RT) domain, and a C-terminal nucleic acid-binding region (including the Thumb, CTI linker, and ZnK zinc finger), along with an endonuclease domain (the RLE nuclease catalytic domain). An N-terminal extension (NTE, located at positions −2 to 0) connects to the RT domain via a linker region. Untranslated regions (UTRs) are present on both sides of the protein-coding sequence. These domains work together to mediate sequence-specific target site recognition, reverse transcription, and site-specific cleavage. **(B)** Three-dimensional schematic of the RNP target site recognition complex. The left panel shows the first-strand DNA nick at the 28 S rDNA target site, generating a 3′-OH that serves as the primer (Red short line) for reverse transcription. The right panel presents the three-dimensional structure: the R2 protein (green) binds specifically to the 28 S rDNA target site through its N-terminal ZnF/Myb domain (blue); the reverse transcriptase domain (RT, orange) catalyzes cDNA synthesis using the R2 RNA template (magenta), forming an RNA-DNA hybrid intermediate; the C-terminal endonuclease domain (RLE, red) performs DNA cleavage; and the 3′ stem-loop and pseudoknot structures of the R2 RNA stabilize the entire ribonucleoprotein complex. This complex enables site-specific recognition, cleavage, and template-guided retrotransposition integration.

The R2 retrotransposon is distinct from other non-LTR retrotransposons as it consists of a single ORF flanked by untranslated regions (UTRs) (Figure 1). This ORF encodes all the proteins required for the retrotransposition process, including a DNA-binding domain responsible for DNA recognition, an RT domain that catalyzes reverse transcription, and an RLE domain that shares homology with restriction endonucleases (Daniels et al., 2025).

### RT domain of R2 retrotransposons

Functioning as the catalytic core, the R2 RT domain possesses distinct enzymatic properties characterized by homology-independent initiation, high processivity, and template jumping. The enzyme can initiate cDNA synthesis using the 3′-end of the DNA target as a primer without requiring sequence homology to the RNA template (Luan and Eickbush, 1995; Luan and Eickbush, 1996; Bibillo and Eickbush, 2002). Once synthesis begins, the RT polymerizes continuously in a single binding event, generating cDNA products 2–5 times longer than those typical of retroviral reverse transcriptases (Bibillo and Eickbush, 2002; Kurzynska-Kokorniak et al., 2007). Such elevated processivity acts as a crucial evolutionary adaptation to compensate for the enzyme’s naturally weak initiation efficiency, ensuring complete transcript synthesis prior to dissociation (Pimentel et al., 2022). Furthermore, upon reaching the end of a template, the RT intrinsically adds non-templated nucleotides to the nascent cDNA. This facilitates template jumping by forming microhomologous base pairs with a newly introduced acceptor template (Bibillo and Eickbush, 2002; Bibillo and Eickbush, 2004).

Structurally, a feature of the R2 retrotransposon is the complete absence of an RNase H domain and its associated degradative activity. Because the original RNA template is not degraded during reverse transcription, the RT domain relies on a robust intrinsic strand-displacement capability to physically displace pre-annealed RNA or DNA strands while transcribing (Jamburuthugoda and Eickbush, 2014; Karin et al., 2017). This specific structural and functional adaptation supports the biochemical model that second-strand synthesis directly utilizes the intact RNA–DNA hybrid as a template (Kurzynska-Kokorniak et al., 2007; Luan et al., 1993; Yang and Eickbush, 1999). Ultimately, this RNase H-independent strand-displacement capacity represents a core functional feature of the RT domain, which may be broadly conserved across non-LTR retrotransposons.

### RLE domain of R2 retrotransposons

The C-terminal region of the R2 retrotransposon contains a RLE domain. *In vitro* biochemical and sequence analyses show that this RLE domain shares similarity with the restriction endonuclease Fok I. Structural comparisons with PD-(D/E)XK nucleases further identify a 95-amino-acid segment in ORF with an αβββαβ fold as the minimal endonuclease domain essential for DNA cleavage (Mukha et al., 2013). Additionally, sequence and structural alignments reveal similarities between the RLE domain and the endonuclease domain of the eukaryotic *Prp8* protein (Mahbub et al., 2017). As for R2, a non-LTR retrotransposon with insertion specificity, this domain is primarily responsible for conferring its targeted integration characteristic (Wilkinson et al., 2023; Kapitonov et al., 2009).

### DNA and RNA binding domains of R2 retrotransposons

The N-terminal region of the R2 retrotransposon harbors a canonical C2H2 zinc finger domain and a Myb-type nucleic acid-binding motif (Yang and Eickbush, 1999). Biochemical evidence indicates that the zinc finger domain interacts with nucleotides 1–3 upstream of the cleavage site, whereas the Myb motif binds approximately 10–15 nucleotides downstream of the insertion site. In addition, the full-length R2 protein protects an extended region spanning 10–40 bp upstream of the insertion site, suggesting the involvement of a C-terminal zinc finger domain in target recognition alongside the N-terminal DNA-binding domains (Christensen et al., 2005). However, mutations in this C-terminal region often cause protein instability, hindering detailed functional analysis (Christensen et al., 2006).

Two key DNA motifs recognized by the R2 protein—RUM (Retrotransposon Upstream Motif) and RASIN (Retrotransposon-Associated INsertion site)—have been identified. Although these sequences occur at multiple locations in rDNA, R2 maintains high insertion specificity (Wilkinson et al., 2023), likely achieved through the coordinated action of its multiple DNA-binding domains, along with contextual factors such as chromatin accessibility. Recent work further reveals that A-clade–specific ZF3–ZF2 domains bind upstream target DNA under specific conditions and modulate second-strand cleavage in proteins such as ZoAl (*Zonotrichia albicollis*) (Lee et al., 2024). Separately, ZnF3–ZF2 has also been reported to bind RNA pseudoknot structures, with ZnF2 specifically recognizing target DNA (Thawani et al., 2025). These findings underscore the functional diversification and evolutionary conservation of N-terminal DNA-binding domains across R2 clades.

The RNA-binding domain, located upstream of the RT domain, contains two conserved motifs, Motif 0 and Motif −1, in which mutations of key residues impair RNA binding, target-primed reverse transcription (TPRT), and second-strand cleavage (Jamburuthugoda and Eickbush, 2014). Structurally related RNA-binding domains are also found upstream of the RT domain in telomerase and group II introns (Rouda and Skordalakes, 2007; Gu et al., 2010; Mitchell et al., 2010). The conservation of motif sequence and spatial organization among these elements supports a common evolutionary origin. In addition, analogous to group II introns, the putative thumb domain of R2 has been proposed as another potential RNA-binding module (Eickbush and Jamburuthugoda, 2008).

In summary, the DNA- and RNA-binding domains of the R2 retrotransposon function not only in substrate recognition but also play essential roles in the retrotransposition mechanism and the evolutionary trajectory of the element.

### The TPRT process of the R2 retrotransposon

**FIGURE 2:**
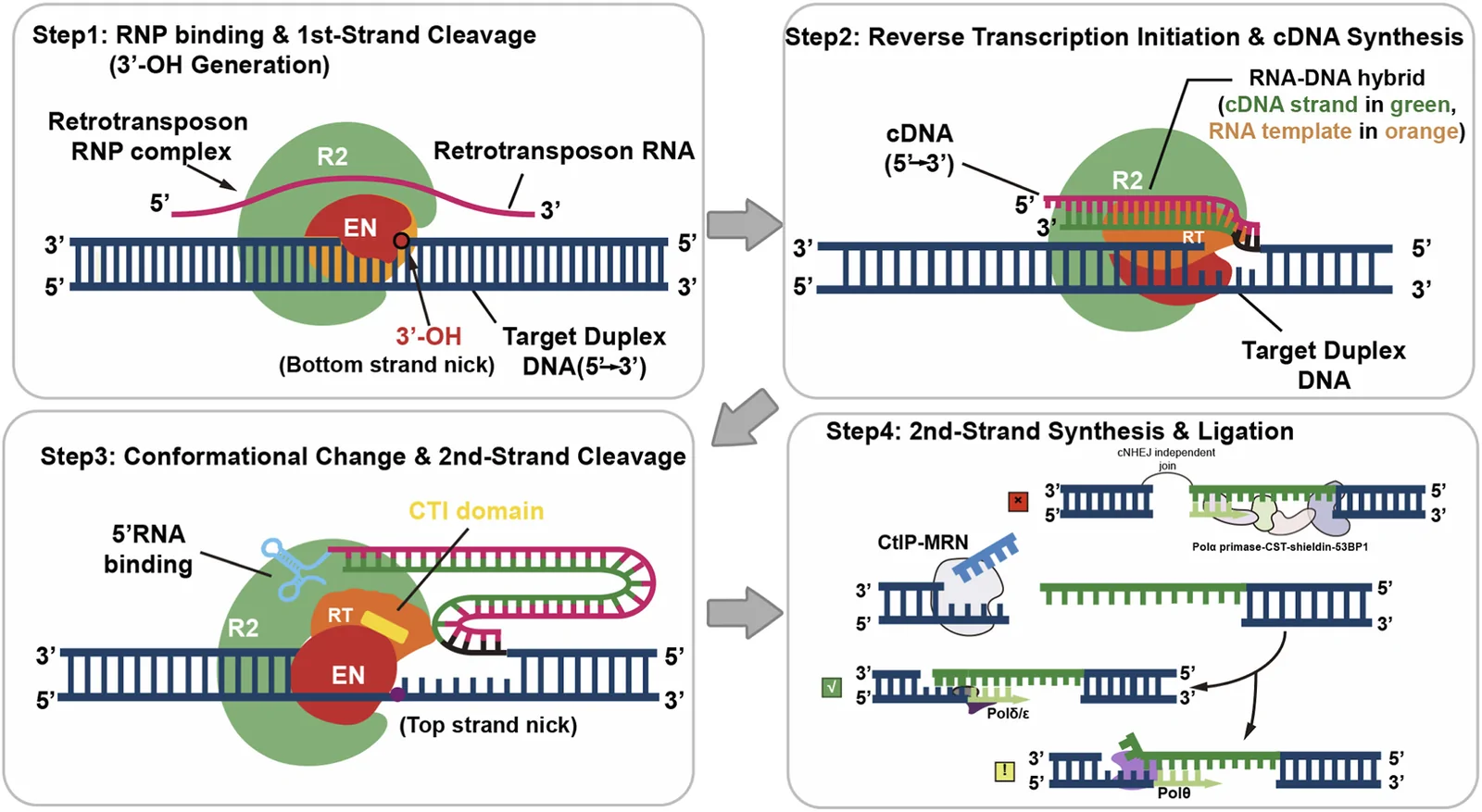
Schematic illustration of the target site-guided retrotransposition integration (TPRT) mechanism of the R2 retrotransposon. The R2 retrotransposon achieves sequence-specific integration through target-primed reverse transcription (TPRT). This process consists of four sequential steps. Step 1: RNP binding to the bottom strand and cleavage (3′-OH generation). The R2 protein (green) and its transcribed RNA copy (megenta) form a ribonucleoprotein complex (RNP). The N-terminal DNA-binding domain specifically recognizes the 28 S rDNA target site (dark blue). The endonuclease domain (EN, red) generates a sequence-specific nick on the bottom strand of the target site, producing a free 3′-OH end (red dot) that serves as the primer for subsequent reverse transcription. Step 2: Reverse transcription initiation and cDNA synthesis. The reverse transcriptase domain (RT) of the R2 protein uses the target site 3′-OH as a primer and the transcribed RNA 3′end (megenta) as a template to extend in the reverse direction, synthesizing complementary DNA (cDNA, green) and forming an RNA-DNA hybrid intermediate. cDNA synthesis proceeds in the 5'→3′direction, extending stepwise along the target site DNA. Step 3: Conformational change and second-strand cleavage. As cDNA synthesis proceeds, the R2 protein undergoes conformational rearrangement. The CTI linker domain (yellow) helps stabilize the complex. The 5′RNA ribozyme region (light blue) may participate in catalysis on the second strand. The endonuclease domain of the R2 protein introduces a second nick on the top strand of the target site (purple dot), preparing the junction for integration. Step 4: Second-strand synthesis and integration. After R2 completes first-strand cDNA synthesis, the host CtIP-MRN complex can initiate end resection to generate single-stranded overhangs. This promotes homologous sequence pairing and allows host Pol δ/ε to synthesize the second strand, enabling precise insertion of a full-length copy (green box). Alternatively, the host may use Pol θ to catalyze second-strand synthesis via microhomology pairing, which usually yields 5′-truncated products (yellow box). When homologous sequences are missing or the above pathways are blocked, the Pol α-primase–CST–shieldin–53BP1 axis steps in. It directly supports primer synthesis and polymerase switching through an alternative end-joining route—either alternative end joining or microhomology-mediated end joining (MMEJ) — that operates independently of cNHEJ, and this route often results in truncations as well (red box). Finally, host ligase then covalently joins the upstream and downstream DNA through the 5′junction point and 3′junction point, completing sequence-specific integration of the R2 copy into the 28 S rDNA target site.

The R2 retrotransposon utilizes a sophisticated TPRT mechanism to achieve site-specific genomic integration and replication (Figure 2).

### Target recognition and first-strand cleavage

R2 retrotransposons initiate insertion through highly selective recognition of the 28 S rDNA locus. This specificity is established by the N-terminal region of the R2 protein, particularly the zinc finger and Myb-containing DNA-binding modules, which engage sequence features flanking the insertion site and position the catalytic endonuclease domain over the correct phosphodiester bond. Structural studies of R2Bm, together with more recent work on R2Tg, have shown that this mode of target recognition differs from that of many other non-LTR retrotransposons, because site selection is not dictated by the endonuclease domain alone but instead depends on coordinated contributions from multiple protein domains and structured RNA elements within the ribonucleoprotein complex (Wilkinson et al., 2023; McIntyre et al., 2025; Thawani et al., 2025; Edmonds et al., 2025; Deng et al., 2023). Once the target DNA is engaged, the restriction-like endonuclease cleaves the bottom strand of the 28 S rDNA target, producing a free 3′-hydroxyl group. This 3′-OH is not simply a break intermediate; it serves immediately as the primer for reverse transcription, thereby directly coupling DNA nicking to cDNA synthesis and ensuring that integration begins only at the cognate chromosomal site (Wilkinson et al., 2023; Christensen and Eickbush, 2005).

### Reverse transcription initiation

After bottom-strand cleavage, the newly exposed 3′-OH primes DNA synthesis by the RT domain using the associated R2 RNA as template. This is the defining step of target-primed reverse transcription. The 3′region of the R2 RNA plays a central role at this stage. Biochemical and structural analyses indicate that the 3′UTR contains conserved secondary structures, including stem-loop and pseudoknot-like elements, that are required for stable RNP assembly and productive initiation of reverse transcription at the target site (Chen et al., 2024; Edmonds et al., 2025; Deng et al., 2023). Earlier mechanistic work had already shown that sequences downstream of the insertion junction strongly influence primer choice and the accuracy of initiation, emphasizing that the RNA template contributes information beyond simple coding capacity (Luan and Eickbush, 1996). In parallel, studies of R2-associated ribozymes demonstrated that the 5′end of the transcript is also structurally and functionally important, because HDV-like self-cleaving ribozymes process the cotranscript and help generate the proper RNA architecture for retrotransposition (Eickbush and Eickbush, 2010; Eickbush et al., 2013; Ruminski et al., 2010). Together, these observations indicate that reverse transcription initiation depends on an integrated RNA-protein scaffold rather than on the reverse transcriptase domain alone.

### Second-strand cleavage and coordinated DNA synthesis

One of the most important advances from recent cryo-EM studies is the realization that second-strand cleavage is temporally controlled rather than occurring simultaneously with the first nick. When the R2 complex is bound to the 3′RNA and target DNA in the pre-initiation state, the machinery is configured to favor bottom-strand cleavage and initiation of first-strand cDNA synthesis while suppressing premature cleavage of the top strand (Wilkinson et al., 2023; Deng et al., 2023). As reverse transcription proceeds, the structure of the complex changes. Work form the *B. mori* reports that release or repositioning of the 3′RNA element, followed by engagement of sequence information from the 5′region of the R2 RNA, promotes a rearrangement that activates top-strand cleavage (Christensen et al., 2006; Edmonds et al., 2025; Deng et al., 2023). Recent engineering-oriented studies have further highlighted that this transition is sensitive to RNA architecture: removal of inhibitory structures in the donor 3′UTR or minimization of unnecessary donor sequences can markedly improve full-length insertion efficiency, presumably by facilitating the later steps of TPRT and second-strand synthesis (Chen et al., 2024). In addition, structural analyses have identified auxiliary protein features, including CTI-associated conformational support, that appear to stabilize the nuclease-active state required for productive progression through the second cleavage step (Wilkinson et al., 2023; Chen et al., 2024).

### Integration completion and repair

Once cDNA synthesis and top-strand cleavage have occurred, the remaining intermediate must be converted into a stable chromosomal insertion. Although the terminal repair steps are still not fully resolved, available evidence indicates that host repair activities complete ligation of DNA ends and synthesis of the complementary strand (McIntyre et al., 2025). In some contexts, imperfect resolution at the 5′junction can lead to extra sequence additions, truncations, or template-jumping events, phenomena that have long been associated with non-LTR retrotransposition and are also discussed in recent R2 engineering studies (Chen et al., 2024; Bibillo and Eickbush, 2004). Nevertheless, when the process proceeds efficiently, R2-mediated integration can achieve a high degree of target specificity and relatively accurate insertion into multicopy rDNA loci, with lower indel formation than might be expected from conventional double-strand break-based editing approaches (Chen et al., 2024; Edmonds et al., 2025). This mechanistic framework is especially important for biotechnology, because it explains why R2 systems can be adapted into all-RNA gene insertion platforms that avoid exogenous donor DNA and reduce the liabilities associated with random integration or broad genomic cleavage. Recent studies in mammalian cells show that, once donor RNA design, protein engineering, and delivery are optimized, R2-based systems can support efficient targeted insertion ranging from reporter-sized payloads to substantially larger cargos, thereby establishing R2 retrotransposons as a promising foundation for future programmable insertion technologies (Fell et al., 2025; Zhang et al., 2024; Edmonds et al., 2025).

### Development of R2 retrotransposon-based gene editing tools

**FIGURE 3:**
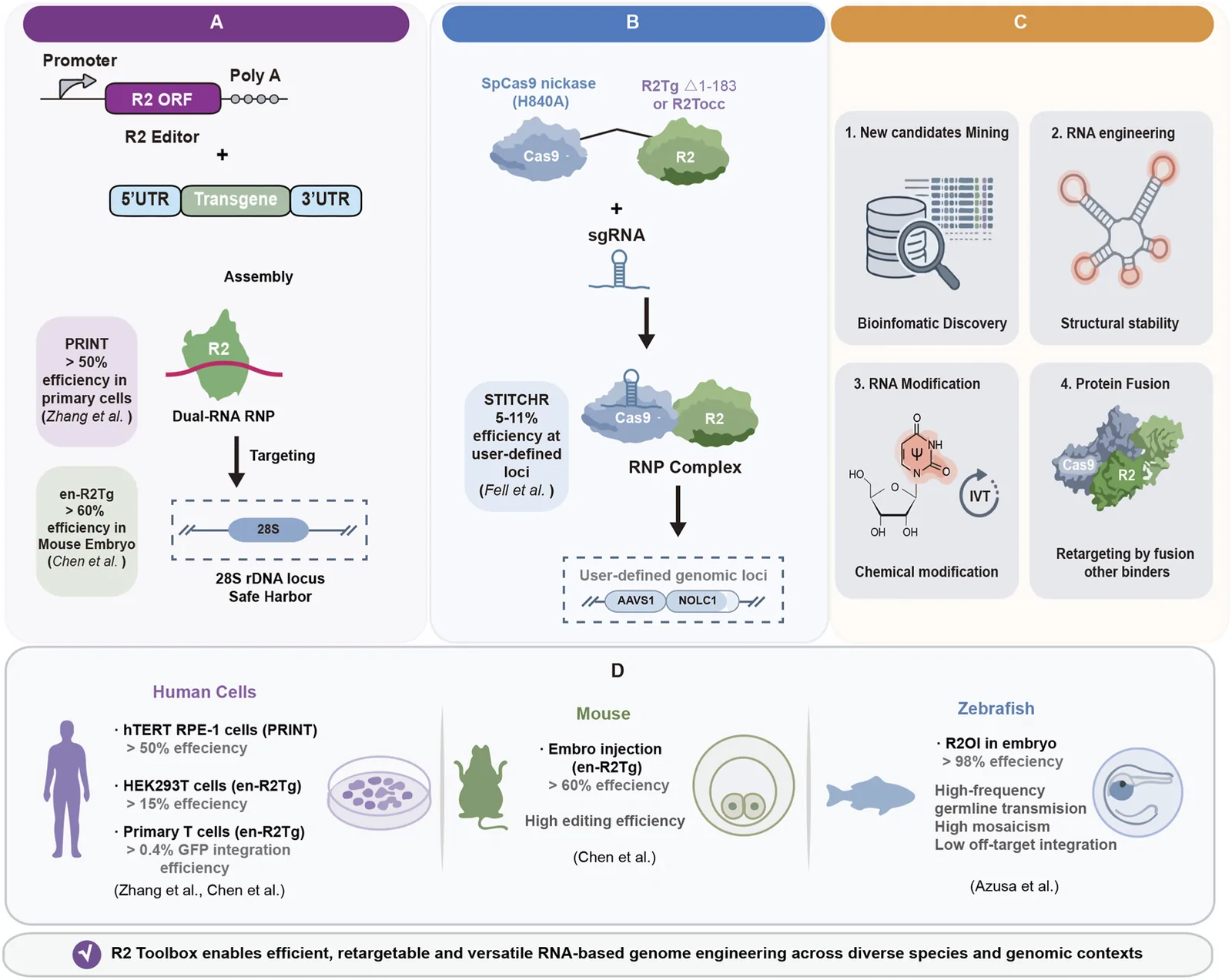
The R2 engineering toolbox: mechanisms, optimization strategies, and multi-species applications. **(A)** Dual-RNA systems (PRINT and en-R2Tg). Here, an R2 protein (the “R2 editor”) assembles with an engineered donor RNA that carries a transgene flanked by the 5′and 3′UTRs. The resulting ribonucleoprotein (RNP) complex targets and integrates the transgene into the conserved 28 S ribosomal DNA (rDNA) locus–a genomic safe harbor. Two examples are highlighted: PRINT achieves over 50% efficiency in primary human cells, while the engineered en-R2Tg reaches over 60% efficiency in mouse embryos. **(B)** Retargeting system (STITCHR). This strategy redirects R2 integration to user-defined loci. STITCHR fuses a catalytically impaired Cas9 nickase (SpCas9 H840 A) to a truncated R2 protein (e.g., R2Tg Δ1-183 or R2Tocc). A standard single-guide RNA (sgRNA) guides the fusion protein to a specific DNA target, where the RNP complex mediates cargo integration. At safe harbor sites such as AAVS1 or NOLC1, reported efficiencies range from 5% to 11%. **(C)** R2 optimization strategies. Four approaches are summarized (Shalem et al., 2015): mining new candidates via bioinformatics to discover R2 orthologs with favorable properties (Wang and Doudna, 2023); engineering donor RNA secondary structure to improve stability and R2 binding (Pacesa et al., 2024); incorporating chemically modified nucleotides (e.g., pseudouridine, Ψ) during in vitro transcription (IVT) to enhance RNA stability and reduce immunogenicity; and (Andrew et al., 2018) fusing the R2 protein with other functional domains (e.g., DNA-binding proteins) to alter targeting specificity or boost activity. **(D)** Multi-species application landscape. R2 tools have been successfully used across different model organisms. In human cells, PRINT and en-R2Tg enable efficient gene integration in hTERT RPE-1, HEK293T, and primary T cells. In mice, direct embryo injection of en-R2Tg components yields >60% editing efficiency. In zebrafish embryos, the R2Ol system shows exceptionally high efficiency (>98%), leading to high-frequency germline transmission with low off-target effects.

Precise, efficient and safe transgene integration remains a central goal in human gene therapy. Since the initial demonstrations of CRISPR/Cas9-based editing, methods such as base editing and prime editing have advanced considerably, yet classical knock-in strategies still depend heavily on homology-directed repair (HDR) for exogenous gene expression (Cong et al., 2013; Doudna and Charpentier, 2014; Hsu et al., 2014; Adli, 2018; Wu et al., 2018; Anzalone et al., 2020). These approaches are often constrained by low recombination efficiency, the considerable burden of donor DNA design, and the risk of unintended genomic disruption (Wang and Doudna, 2023; Andrew et al., 2018; Pickar-Oliver and Gersbach, 2019). In this context, site-specific non-LTR retrotransposons—particularly R2 elements—have drawn increasing interest as an alternative integration platform (Figure 3).

### R2 retrotransposons as a platform for targeted genomic integration

The R2 retrotransposon integrates specifically into the 28 S rDNA locus, a multicopy genomic region previously characterized as a safe harbor for exogenous gene insertion. Located outside protein-coding genes, this locus causes minimal disruption to endogenous gene expression (Lisowski et al., 2012; Yl-Herttuala, 2020). Unlike commonly used genomic safe harbors such as AAVS1 and ROSA26—which reside in gene-rich regions proximal to oncogenes—the 28 S rDNA locus is present in hundreds of copies per vertebrate genome. Moreover, the approximately 300 bp sequences flanking the R2 insertion site are strongly conserved across vertebrates, including humans, providing a consistent and reliable target for engineering (Kuroki-Kami et al., 2019; Su et al., 2019).

The first R2-based site-specific integration in human cells was achieved using R2Ol, a retrotransposon identified from medaka fish (*Oryzias latipes*). Delivered via an adenovirus-based system into human 293 T cells, both the 3′and five ends of R2Ol inserted at the correct 28 S rDNA target position, with no detectable off-target integration. Using a trans-complementation strategy—a helper adenovirus expressing the R2Ol protein co-delivered with a donor plasmid carrying an EGFP cassette—exogenous gene integration into the 28 S target was successfully achieved. This represented the first LINE-based sequence-specific gene knock-in system in human cells (Su et al., 2019). Full-length insertion of sequences exceeding 4.2 kb was confirmed, establishing R2Ol as capable of accommodating larger therapeutic cargos and laying the foundation for subsequent engineering efforts in mammalian systems.

### PRINT and en-R2Tg: establishing all-RNA delivery systems for targeted transgene insertion

Because R2-based integration proceeds from an RNA template rather than an exogenous DNA donor, these systems are inherently compatible with non-viral RNA delivery platforms such as lipid nanoparticles (LNPs). They also avoid the immunogenicity and random integration risks often associated with DNA-based approaches. Two studies established functional all-RNA gene insertion systems based on this principle. Despite following distinct engineering strategies, both approaches demonstrated the feasibility and efficiency of R2-based transgene delivery (Zhang et al., 2024; Chen et al., 2024).

PRINT uses two *in vitro* transcribed RNAs co-delivered into human cells: messenger RNA encoding the R2 protein and template RNA carrying the transgene payload. Systematic screening identified ZoAl as the most active R2 ortholog for this purpose, and the final system supported targeted insertion of transgenes up to 4 kb in length. More than 50% of primary human cells acquired stable transgene copies, over half of which were full-length, with an estimated error rate of approximately one per 10,000 bp (Zhang et al., 2024). The en-R2Tg system adopted a more explicitly engineering-driven approach, combining R2 ortholog screening with protein engineering—including targeted amino acid substitutions and domain insertions—along with donor RNA redesign. Collectively, these modifications raised integration efficiency to over 20% in human cell lines under plasmid-based conditions and above 60% in mouse embryos under all-RNA delivery. LNP-mediated delivery across multiple cell types, including HEK293 T (15.6%), Huh7 (24.8%), N2a (12.8%) cells and human primary T cells, confirmed broad applicability; high-throughput sequencing established an on-target specificity of approximately 99% (Chen et al., 2024). Further structural and engineering work by Edmonds and colleagues produced a compact donor design incorporating chemical modification at the donor RNA 5′end, pushing integration efficiency beyond 80% in several human cell lines (Edmonds et al., 2025). Collectively, these studies demonstrate that all-RNA R2-based integration can achieve clinically meaningful efficiency levels across diverse mammalian cell types.

### STITCHR: programmable retargeting beyond the 28S locus

A limitation of the above systems is that they only target the endogenous 28 S rDNA locus. Redirecting R2 integration to user-defined genomic sites would expand the therapeutic potential of these tools. One recent study addressed this issue by performing a large-scale computational screen of 4,464 animal genome assemblies, identifying multiple site-specific retrotransposon families with distinct integration preferences (Fell et al., 2025). Certain R2 orthologs showed natural reprogrammability: their target preference could be redirected by modifying the homology arms of the template RNA without disrupting the core integration activity.

Among the characterized orthologs, R2Tocc was selected for further development due to its high reprogramming efficiency and strongly reduced residual integration at the native 28 S site—an important safety feature for therapeutic use. Fusing SpCas9^H840A^ to a truncated R2Tocc generated the STITCHR system (site-specific target-primed insertion via targeted CRISPR homing of retroelements). In this system, the Cas9 nickase directs the complex to user-defined loci, while the R2 component performs template-directed insertion (Fell et al., 2025). STITCHR has been tested in a range of editing applications, including single-base substitutions, small insertions, therapeutic cargo delivery, and large gene insertions of clinically relevant genes such as *BTK*, *CEP290*, *HBB*, *HEXA*, *OTC* and *PAH*, as well as synthetic sequences up to 12.7 kb. The system is compatible with *in vitro* transcription and synthetic RNA templates, supports multiplexed insertion, and functions in both dividing and non-dividing cells. Minimal indel formation at target sites was confirmed using a validated three-primer next-generation sequencing assay and droplet digital PCR.

### Comparative advantages and limitations of R2-based tools

R2-based integration tools differ from established platforms in several practically relevant respects (see Table 1). Lentiviral vectors deliver high transduction efficiency (50%–90% *in vitro*) but insert semi-randomly into actively transcribed genomic regions, a property associated with insertional mutagenesis and oncogene activation in clinical settings (Schröder et al., 2002; Kumar et al., 2001). AAV vectors are predominantly episomal and subject to a strict payload ceiling of approximately 5 kb, limiting their use for larger therapeutic transgenes (Naso et al., 2017; Samulski and Muzyczka, 2014; Mittas et al., 2026). Prime editing circumvents DSBs and achieves indel rates below 0.5% in optimized configurations, yet its practical utility is largely confined to corrections below 100 bp (Chen and Liu, 2022). TwinPE has no theoretical size limit, with efficiencies from 5% to 40% depending on spacer length. But it requires multi-vector delivery, and the dual pegRNAs can cause off-target cuts (Anzalone et al., 2022). PASTE handles 5–36 kb inserts but works at only 1%–10% (in bacteria). It is integrase-based and site-specific, yet transposon hopping, rearrangements, and oncogenesis are real concerns. Cargo limits and complex regulation do not help either (Yarnall et al., 2023). R2/PRINT and en-R2Tg avoid these issues by operating entirely through RNA components, without viral vectors or exogenous DNA. Insertion capacity has been experimentally confirmed up to approximately 2 kb, on-target specificity reaches approximately 99%, and indel formation remains below 0.1% (Zhang et al., 2024; Chen et al., 2024; Edmonds et al., 2025). STITCHR extends these capabilities further, supporting cargo sizes up to 12.7 kb and enabling integration at user-defined loci beyond the 28 S rDNA site, with efficiencies of 5%–11% across multiple genomic targets and minimal off-target activity (Fell et al., 2025).

**TABLE 1:** Comparison of the R2 retrotransposon-based gene integration tool with existing platforms.

Technology (Ref)	Max insertion size	Editing efficiency (typical value)	DSB dependency	Integration mode	Primary off-target risk	Delivery vector constraints
R2/PRINT Zhang et al. (2024); Chen et al. (2024)	enR2Tg: ∼2 kb PRINT: ∼4 kb (experimentally confirmed)	HEK293 T (after en-R2Tg optimization): ∼20%; mouse embryo ∼60%; LNP delivery: 5%–15%	No	Site-specific integration into 28 S rDNA	Extremely low indels (<0.1%); recombination between rDNA multi-copy loci; 5′truncated integration products	Pure RNA delivery (low cost/easy scale-up); increase cargo size reduces full-length integration efficiency; requires optimization of the LNP/mRNA delivery system
R2/STITCHR Fell et al. (2025)	Experimentally verified: 12.7 kb	AAVS1 locus: ∼10%; NOLC1 locus: 1%–2%; multiple knock-in: 6%–12%	No	Cas9-guided site-specific integration into any locus (e.g., AAVS1, NOLC1)	Cas9-mediated off-target cleavage; extremely low off-target rDNA integration; higher fidelity than HDR	Requirement for dual-plasmid/aav co-delivery; SpCas9-R2 fusion protein >200 kDa
Prime editing Anzalone et al. (2019)	<100 bp (high efficiency), up to ∼1 kb	HEK293 T cells: optimized PE: up to 90% efficiency, vPE (low indels): 40%–70%; in vivo: <10%	No (single-strand nick dependent)	*In situ* editing	pegRNA-mediated indels (PE2: 1%–5%, vPE: <0.5%); unintended deletions from nick dependent; low-frequency off-target editing (target: Off-target >1000:1)	PE protein >170 kDa (difficult for AAV delivery), requires dual-aav/lnp vectors; pegRNA stability limitations
TwinPE Anzalone et al. (2022)	Theoretically no upper limit	5%–40% (depends on spacing)	No	Targeted deletion/inversion	Accumulation of off-target cuts from dual pegRNAs	Multi-vector delivery required for dual-PE systems
PASTE Yarnall et al. (2023)	∼5–36 kb	1%–10% (in bacteria)	No (dual-nick strategy)	Integrase-mediated site-specific integration	Non-specific transposon hopping; risk of chromosomal rearrangements; insertional mutagenesis oncogenesis risk	Transposon cargo limitations; complex expression regulation
Lentivirus Kumar et al. (2001)	∼8–10 kb (effective payload ∼6 kb)	*In vitro* transduction: 50%–90%; in vivo liver: 10%–30%	Yes (integration requires reverse transcription + integrase)	Semi-random integration (prefers active transcription regions)	Insertional mutagenesis oncogenesis risk; transcriptional activation/silencing; genomic rearrangements	Moderate immunogenicity; production titer limits (10^8^–10^9^ TU/mL); serotype restricts tissue tropism
AAV Naso et al. (2017)	∼4.7 kb (effective <4.5 kb with ITRs)	Liver: 30%–80% (AAV8/9)	No	Primarily episomal; very low probability (<0.1%) of random integration at AAVS1 locus	Neutralizing antibody induction; genomic DNA contamination	Strict size limit (>5 kb significantly reduces efficiency); pre-existing immunity limits redosing; high production cost (GMP grade 10^13^ vg/dose)

Several limitations nevertheless remain. Under LNP delivery conditions, full-length integration efficiency typically falls in the 5%–15% range and declines further as cargo size increases—a constraint with direct implications for transgenes of therapeutic scale. STITCHR requires co-delivery of a SpCas9–R2 fusion protein exceeding 200 kDa, a packaging burden comparable to that encountered with prime editing constructs (Anzalone et al., 2020). The long-term stability of transgenes integrated at rDNA loci in proliferating cells has yet to be systematically characterized, and this question will need to be addressed before clinical application becomes feasible. These are genuine technical obstacles rather than fundamental incompatibilities, and ongoing advances in RNA engineering, delivery optimization, and ortholog diversification suggest tractable paths forward. Taken together, the current profile of R2-based tools—DSB-independent, site-specific, and compatible with scalable RNA-only delivery—positions them as a substantive and complementary option within the broader gene integration landscape.

## Discussion

The R2 retrotransposon mediates RNA-directed, site-specific integration via target-primed reverse transcription (TPRT). Unlike knock-in strategies based on homology-directed repair (HDR), TPRT does not require double-strand breaks (DSBs) or the HDR machinery. Instead, it directly uses the 3′-OH generated by site-specific cleavage of the genomic target as the primer for reverse transcription. It then synthesizes cDNA from the R2 RNA template to complete integration. This mechanism bypasses the intrinsic limitations of HDR, such as low efficiency in non-dividing cells and constrained donor template design. Nevertheless, the details of second-strand DNA synthesis and the completion of integration remain unclear. Recent cryo-EM structures have captured the conformational states of the R2-RNP complex (from A-clade) during TPRT and after second-strand nicking (Thawani et al., 2025). However, several aspects remain to be investigated: the protein conformational changes during later integration steps, how host DNA polymerase and ligase are recruited, and the structural basis for 5′-truncated product formation.

Under LNP delivery conditions, the efficiency of full-length integration typically ranges from 5% to 15% and declines with increasing cargo length (Chen et al., 2024). Moreover, the N-terminal DNA-binding domain of R2 is highly specific for the 28 S rDNA sequence. This specificity underpins the system’s extremely low off-target activity, but also represents the main limitation on its application scope. The STITCHR system uses a SpCas9-R2 fusion protein and achieves targeted integration into user-defined loci such as AAVS1 and NOLC1 with efficiencies of 5%–11% (Fell et al., 2025). However, this design inevitably introduces Cas9-associated off-target cleavage risks. In addition, the fusion protein is over 200 kDa, which adds challenges for RNA delivery. A potential alternative is to rationally design the R2 DNA-binding domain or evolve it through directed evolution to recognize conserved sequences in endogenous safe harbor sites (e.g., AAVS1, ROSA26). This approach is similar to the retargeting of piggyBac transposases via zinc finger or TALE fusions (Owens et al., 2013). This would extend R2’s targeting ability while preserving its core catalytic function. It could also provide a basis for developing orthogonal R2 variants that recognize different genomic loci.

The diversity of these tools and their clinical feasibility are equally important for advancing this technology toward application. Given the vast diversity in nature, R2 is likely only one representative among many specific retrotransposon families. Although R2 elements have been identified in at least six animal phyla (Kenji and Kojima, 2004; Jakubczak and Eickbush, 1991; Kojima and Haruhiko, 2005; Kojima et al., 2006; Besansky et al., 1992), only a subset of these orthologs possess the biochemical properties suitable for engineering into genome editing tools. Since the R2 retrotransposon from Oi was reported to integrate in zebrafish and human cells, several other R2 retrotransposons have also been shown to integrate in human cells, including R2Bm, R2GFo, R2Tg, R2ZoAl, and R2TOcc (Deng et al., 2023; Fell et al., 2025; Zhang et al., 2024; Chen et al., 2024; Edmonds et al., 2025). Recent engineering studies have deliberately chosen different R2 orthologs to exploit their distinct functional features. For example, R2Bm (clade D) from the silkworm *B. mori* has been the main model for structural studies due to its stability and the availability of high-resolution structural data (Wilkinson et al., 2023; Deng et al., 2023). In contrast, ZoAl (from the white-throated sparrow *Z. albicollis*) was identified in a high-throughput screen as the variant with the highest activity in human cells and was therefore chosen for the PRINT system (Zhang et al., 2024; Lee et al., 2024). Similarly, en-R2Tg used R2Tg (from the zebra finch *Taeniopygia guttata*) because of its compact size and ease of protein engineering, including a truncated C-terminal inhibition (CTI) domain (Chen et al., 2024; Thawani et al., 2025; Edmonds et al., 2025). Finally, the STITCHR system used R2Tocc, an ortholog found through bioinformatic analysis (Fell et al., 2025). This ortholog was selected for its high reprogrammability and reduced residual activity at the endogenous 28 S locus—a feature critical for safety in retargeting applications.

Therefore, systematic mining and identification of more R2-like retrotransposons with distinct targeting specificities will significantly expand our toolkit and provide diverse options for tackling complex gene therapy scenarios. Ultimately, all foundational research and technological development efforts must be rigorously validated for function and safety. This validation should be performed in relevant *in vitro* cell models and *in vivo* animal disease models, such as those for genetic disorders and cancer. This step is critical, as it will assess therapeutic efficacy, long-term stability, and potential immunogenicity in realistic pathological contexts. Such assessment will clear a path for clinical applications in human gene therapy and lay a solid scientific foundation.
